# Aspirin inhibit platelet-induced epithelial-to-mesenchymal transition of circulating tumor cells (Review)

**DOI:** 10.3892/br.2014.242

**Published:** 2014-02-27

**Authors:** XIAO-LIANG LOU, JUN DENG, HUAN DENG, YUAN TING, LV ZHOU, YAN-HUA LIU, JIN-PING HU, XIAO-FENG HUANG, XIAO-QING QI

**Affiliations:** 1 Department of Neurology, The Fourth Affiliated Hospital of Nanchang University, Nanchang, Jiangxi 330003, P.R. China; 2 Emergency Department, The First Affiliated Hospital of Nanchang University, Nanchang, Jiangxi 330006, P.R. China; 3 Department of Pathology, The Fourth Affiliated Hospital of Nanchang University, Nanchang, Jiangxi 330003, P.R. China; 4 Renmin Institute of Forensic Medicine in Jiangxi, Nanchang, Jiangxi 330003, P.R. China

**Keywords:** aspirin, metastasis, platelets, epithelial-to-mesenchymal transition, circulating cancer cells

## Abstract

Metastasis, a cascade of events beginning with epithelial-to-mesenchymal transition (EMT), is the main cause of cancer-related mortality. EMT endows circulating cancer cells (CTCs) with invasive and anti-apoptotic properties. These transitioning cells leave the primary tumor site and travel through the circulation to populate remote organs, even prior to the onset of clinical symptoms. During this journey, CTCs activate platelets, which in turn secrete α-granules. These α-granules contain high levels of transforming growth factor-β (TGF-β) and platelet-derived growth factor (PDGF), both considered to be powerful activators of EMT. Recently, regular aspirin use was associated with a reduced risk of cancer metastasis. However, the molecular mechanism underlying the chemotherapeutic effects of aspirin on metastasis has not been fully elucidated. As platelets lack a nucleus, regular aspirin use may exert long-lasting effects on irreversible inhibition of cyclooxygenase (COX)-1 and, subsequently, the secretion of α-granules, which contributes to the maintenance of the EMT state of CTCs. Thus, we hypothesized that the inhibition of platelet-induced EMT of CTCs through the COX-1 signaling pathway may contribute to the intriguing antimetastatic potential of aspirin.

## 1. Introduction

An estimated 90% of cancer deaths are the result of metastasis. Therefore, elucidating the mechanisms involved in this process is crucial. Metastasis is considered to begin with epithelial-to-mesenchymal transition (EMT), a cascade of events during which tumor cells lose their epithelial characteristics and acquire mesenchymal cell characteristics (1). The change in the tumor cells is accompanied by an increase in motility and matrix invasion. Once the malignant cells become detached from the primary tumor site and enter the bloodstream or lymphatic vessels, they become circulating tumor cells (CTCs). Several patients with early-stage cancer have a poor prognosis, since CTCs may reach a secondary organ prior to the onset of clinical symptoms. To exploit the window of opportunity for therapeutic intervention between initial dissemination and eventual metastatic recurrence, a better understanding of the biological behavior of CTCs is required.

## 2. CTCs and EMT

EMT, a transient and reversible process, is considered to enhance the capacity of cancer cells to invade, access the vasculature, metastasize and resist apoptosis (2). Primary tumors may recruit various cells into their microenvironment and secrete transforming growth factor-β (TGF-β), which is considered to be the most potent inducer of EMT. EMT promotes a patchy asynchronous development that involves relatively small numbers of primary cancer cells (3). These transitioning cancer cells then acquire an invasive phenotype and translocate from the primary tumor site to the vasculature (4). However, the microenvironment of CTCs is clearly different from their primary counterpart and there is currently some debate regarding whether EMT is involved in the biological events of CTCs.

Accumulating evidence indicates that CTCs share many morphological and phenotypical traits with cells undergoing EMT (5). The majority of CTCs obtained from the peripheral blood of patients with breast or prostate cancer co-express epithelial and mesenchymal markers, including E-cadherin, cytokeratin (CK), vimentin and N-cadherin (6,7). EMT-related antigens are also found in CK^−^/CD45^−^ cells, suggesting that these cells may represent CTCs that have undergone complete EMT (8,9). Inhibition of pivotal elements in EMT-associated signaling pathways, such as Twist1, Zeb1, Zeb2, SNAIL1 and SNAIL2/Slug, has been associated with a decreased risk of metastatic relapse (10). However, the molecular mechanisms by which CTCs maintain the EMT state have not been elucidated.

## 3. Platelets promote EMT of CTCs

Thrombocytosis is observed in several metastatic cancers and correlates with a worse prognosis, indicating that platelets play a significant role in cancer metastasis (11). In addition to their well-established role in protecting CTCs against mechanical and immune assaults in the circulation, platelets were recently shown to induce EMT in CTCs (12). In addition, platelets are activated through direct interactions with CTCs and secrete α-granules, which contain TGF-β and platelet-derived growth factor (PDGF) at concentrations several-fold higher compared to that in most cell types (13). Treatment with platelets induces increased phosphorylation of the TGF-β signaling effector Smad2 and Smad-binding element-dependent transcription (12). Platelet-secreted PDGF is another important mediator of EMT. Overexpression of PDGF-D, a member of the PDGF family, in prostate cancer cells promotes EMT *in vitro* and *in vivo* through the activation of the mammalian target of rapamycin downstream targets S6K and 4E-BP1 (14). PDGF-D may also increase the expression of Notch-1 in pancreatic cancer cells, which is known as a conserved ligand receptor pathway and an inducer of EMT (15). The extensive crosstalk between PDGF-D and multiple signaling pathways, such as nuclear factor κ-light-chain-enhancer of activated B cells, chemokine (C-X-C motif) receptor 4 and B-cell lymphoma 2 pathways, suggest that efficient inhibition of PDGF during EMT may prevent the progression of metastasis (16–18). Another study indicates that autocrine platelet-derived growth factor receptor (PDGFR) signaling may contribute to the maintenance of EMT, possibly through activation of the signal transducer and activator of transcription (STAT) 1 (19).

In addition to platelet-derived PDGF, a previous study revealed that TGF-β signaling may increase the expression of PDGF in cancer cells, which acts in a sequential auto- or paracrine manner to promote sustained EMT (20). The components of the PDGF signaling pathway were found to upregulated during TGF-β-induced EMT in breast cancer (21). The TGF-β-inducible secretion of interleukin-like EMT-inducer may upregulate the expression of PDGF and PDGFR, leading to signaling via β-catenin and STAT3 to establish EMT (22). TGF-β-induced PDGF activates phosphatidylinositol-3 kinase and, furthermore, increases the accumulation of nuclear β-catenin (23). In gliomas, high TGF-β signaling is associated with a poor prognosis and promotes glioma cell proliferation by activating PDGF-B/PDGFR signaling (24). Based on the abovementioned findings, we may reasonably deduce that cytokines released by activated platelets contribute to the EMT of CTCs.

## 4. Chemotherapeutic effects of aspirin

Accumulating evidence from observational studies in humans indicates that aspirin reduces the incidence of colorectal cancer and increases the overall survival of cancer patients after a delay of 8–10 years (25–27). One hypothesis argues that aspirin inhibits the malignant transformation from adenoma to adenocarcinoma and this process may take a long time. However, recently published meta-analyses of the results from randomized trials provided evidence that daily aspirin treatment at doses of ≥75 mg reduced all-cancer mortality after only 5 years (27,28). Those results can hardly be interpreted by aspirin only affecting carcinogenesis or early cancer growth. Aspirin was recently shown to improve the prognosis of metastatic cancer patients with unknown primary site (28). In a separate analysis of five randomized trials in the UK on daily aspirin use at ≥75 mg, the risk of cancer with distant metastases was also reduced (29). These accumulating data suggest that aspirin may act as an inhibitor of cancer metastasis. The molecular mechanism that defines aspirin and other non-steroidal anti-inflammatory drugs as a class, is their ability to block the prostaglandin H or the cyclooxygenase (COX) pathway. Inhibition of COX activity decreases the formation of prostanoids, including PGD2, PGE2, PGF2α, PGI2 and thromboxane (TXA) 2 (30). TXA2 is a major metabolite in platelets that promotes their activation and aggregation and, in turn, release of their α-granules. COX-1 is the only isoform present in mature platelets. Aspirin irreversibly inactivates COX-1 through selective acetylation of a critical serine residue within the COX-channel (Ser529). Therefore, the chemotherapeutic effects of aspirin on the metastatic process may depend on the inhibition of platelet-related COX-1 signaling pathway.

## 5. Hypothesis and implications

**Figure 1:**
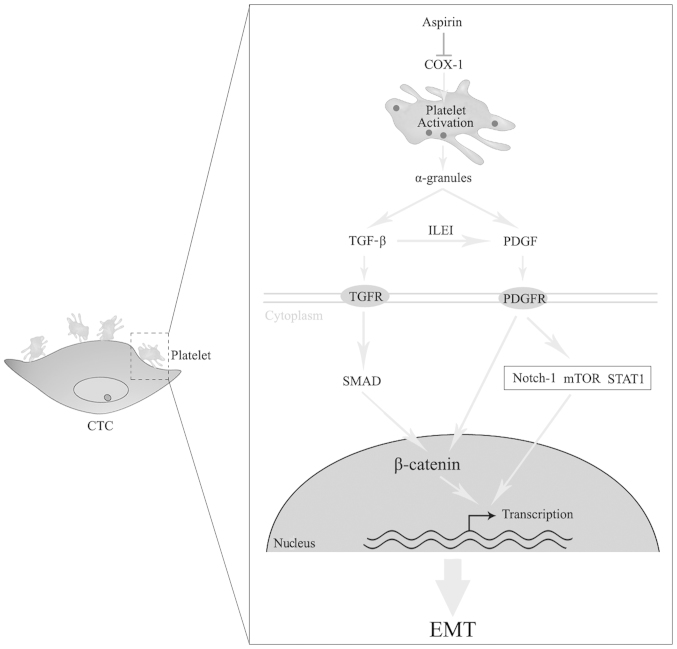
The direct interaction between circulating tumor cells (CTCs) and platelets may promote the activation and aggregation of platelets, which in turn secrete α-granules to upregulate epithelial-to-mesenchymal transition (EMT)-associated signaling pathways, such as transforming growth factor-β (TGFβ) and platelet-derived growth factor (PDGF) pathways. Aspirin irreversibly inhibits the activity of platelet-related cyclooxygenase (COX)-1 and the subsequent formation of thromboxane A2. ILEI, interleukin-like EMT-inducer; TGFR, TGF receptor; PDGFR, PDGF receptor; CTC, circulating cancer cell; mTOR, mammalian target of rapamycin; STAT, signal transducer and activator of transcription.

Based on abovementioned data, we hypothesized that the downregulation of the platelet-related COX-1 pathway may contribute to the antimetastatic effects of aspirin through inhibiting the EMT of CTCs (Fig. 1). The platelet-tumor cell interactions are transient and occur only within the first 24 h (31). Activated platelets may provide a pulse of TGF-β and PDGF, which in turn promotes CTCs to undergo EMT. The recovery of COX-1 activity after treatment with aspirin requires *de novo* synthesis of this enzyme. Platelets lack a nucleus, thus low-dose aspirin (75–162.5 mg) treatment may exert a long-lasting effect on the inhibition of COX-1-related EMT. As the dissemination of CTCs may occur during the early stages of cancer, preventive aspirin use may provide significant therapeutic benefits.

The most frequently reported severe adverse event associated with regular aspirin use is gastrointestinal bleeding. Previous studies reported that the incidence of this adverse event is largely dose-related, with the risk of bleeding being generally higher with standard-dose (300–325 mg) compared to that with low-dose aspirin (75–162.5 mg) (32–34). Therefore, the benefits of long-term use of low-dose aspirin for the prevention of cancer metastasis may outweigh the consequences associated with the increased risk of bleeding.

Cancer metastasis is commonly encountered and is associated with severe clinical consequences that arise from the formation of CTCs. However, the currently available treatments are insufficient for the effective management of these disorders. Therefore, the characterization of the biological behavior of CTCs is crucial in manipulating this process therapeutically. Aspirin may represent an anticancer drug for modulating the platelet-related EMT of CTCs. Should our hypothesis be confirmed, it may change the way we treat metastatic cancer.
